# Supplementary material for: Atypical meiosis can be adaptive in outcrossed Schizosaccharomyces pombe due to wtf meiotic drivers
Source: eLife. 2020 Aug 13;9:e57936. doi: 10.7554/eLife.57936 (PMC7426094; doi:10.7554/eLife.57936)
Supplement: Supplementary file 1. — #Strains SZY1535 and SZY1537 are Ura+ because when the Sk wtf4 allele was originally made (described in Nuckolls et al., 2017), the strains retained the ura4+ cassette. The ura4 cassette is at an unknown location but linked to the wtf4 locus. *Strains SZY3910 and SZY3911 were generated via crossing. One of the parental strains had a wild-type ura4 allele and was thus Ura+. The other parental strain contained the ura4-D18, but had a rec12∆::ura4+, and thus was also Ura+. However, when we crossed the strains to generate SZY3910 and SZY3911, we confirmed the presence of the rec12 allele using PCR, but we did not determine if they contained the ura4-D18 or the ura4+ allele. [file elife-57936-supp1.docx]

| **Strain** | **Other name** | ***S. pombe* isolate** | **Genotype** | **Reference** |
| --- | --- | --- | --- | --- |
| CBS5680 | SZY2050 | CBS5680 | Wild type |  |
| JB916 | SZY1150 | FY29033 | Wild type |  |
| JB844 | SZY2042 | JB844 | Wild type |  |
| JB873 | SZY2044 | JB873 | Wild type |  |
| JB929 | SZY2055 | JB929 | Wild type |  |
| JB939 | SZY2045 | JB939 | Wild type |  |
| JB1172 | SZY2047 | JB1172 | Wild type |  |
| NBRC0365 | SZY2056 | NBRC0365 | Wild type |  |
| SZY44 | GP745 | *Sp* | *h-, lys4-95* | Zanders et al 2014 |
| SZY47 |  | *Sk* | *h90,* *ura4*∆*::kanMX4* | Zanders et al 2014 |
| SZY122 |  | *Sk* | *h90, rec12*∆::*ura4+, ura4*∆::*kanMX4,* *lys4*∆::*kanMX4* | Zanders et al 2014 |
| SZY180 |  | *Sk* | *h90,* *lys1*∆::*kanMX4* | Zanders et al 2014 |
| SZY186 |  | *Sk* | *h90,* *ade6*∆::*hphMX6* | Zanders et al 2014 |
| SZY196 |  | *Sk* | *h90, rec12*∆*::ura4+, ura4*∆*::kanMX4, lys1*∆*::kanMX4* | Zanders et al 2014 |
| SZY197 |  | *Sk* | *h90,* *ura4*∆::*kanMX4*, *lys1*∆::*kanMX4* | Zanders et al 2014 |
| SZY201 |  | *Sp* | *h-, lys1-37, rec12-171::ura4+, ura4-x (ura4-294 or ura4-D18)* | Zanders et al 2014 |
| SZY208 |  | *Sk* | *h90, ade6*∆*::hphMX6, his5*∆*::natMX4, ura4*∆*::kanMX4, rec12*∆*::ura4+* | Nuckolls et al 2017 |
| SZY293 |  | *Sk* | *h90, ura4*∆*::kanMX4, ade6*∆*::hphMX6, rec12*∆*::ura4+* | Zanders et al 2014 |
| SZY294 |  | *Sk* | *h90, ura4*∆*::kanMX4, his5*∆*::natMX4, rec12*∆*::ura4+* | Zanders et al 2014 |
| SZY296 |  | *Sk* | *h90, ura4*∆*::kanMX4, lys1*∆*::kanMX4, ade6*∆*::hphMX6, rec12*∆*::ura4+* | Zanders et al 2014 |
| SZY297 |  | *Sk* | *h90,* *ura4*∆::*kanMX4*, *lys1*∆::*kanMX4*, *ade6*∆::*hphMX6* | Zanders et al 2014 |
| SZY320 |  | *Sk* | *h90, ura4*Δ*::natMX4* | Zanders et al 2014 |
| SZY480 | GP282 | *Sp* | *h-, his5-303* | from Gerry Smith's lab |
| SZY580 |  | hybrid with *Sk* karyotype | hybrid chr3, *Sk* chr1 and chr2. *h90, rec12*∆*::ura4+, his5*∆*::natMX4, ura4-294* | this work |
| SZY643 |  | *Sp* | *h90, leu1-32, ura4-D18, wtf18-2* | Nuckolls et al 2017 |
| SZY661 |  | *Sk* | *h90, ura4*Δ*::natMX4, leu1*Δ*::hphMX6* | Nuckolls et al 2017 |
| SZY748 |  | *Sk* | *h90, ura4*Δ*::kanMX4, ade6*Δ::*hphMX6* | Zanders et al 2014 |
| SZY790 |  | *Sp* | *h-, his5-303, rec12-169::3HA6His-kanMX6, ade6*∆*::hphMX6* | this work |
| SZY791 |  | *Sp* | *h-, his5-303, rec12-169::3HA6His-kanMX6, ade6*∆*::hphMX6* | this work |
| SZY887 |  | *Sp* | *h90, leu1-32, ura4-D18, ade6-::Sk wtf4::kanMX4::ade6-* | Nuckolls et al 2017 |
| SZY925 |  | *Sp* | *h90, leu1-32, ura4-D18, ade6-::kanMX4::ade6-* | Nuckolls et al 2017 |
| SZY969 |  | *Sp* | *h90, leu1-32, ura4-D18, ade6-::Sk wtf4:hphMX6::ade6-* | Nuckolls et al 2017 |
| SZY1051 |  | *Sp* | *h90, leu1-32, ura4-D18,*  *ade6-::Sk wtf4(M1X,M12X):kanMX4::ade6-* | Nuckolls et al 2017 |
| SZY1151 |  | *Sp* | *h-, lys4-95, ade6-::Sk wtf28:kanMX4::ade6-* | Nuckolls et al 2017 |
| SZY1178 |  | *Sp* | *h90, leu1-32, ura4-D18, ade6-::hphMX6::ade6-* | this work |
| SZY1180 |  | *Sp* | *h-, lys4-95, ade6-::hphMX6::ade6-* | Bravo Núñez et al 2020 |
| SZY1293 |  | *Sp* | *h-, lys4-95, ade6-::Sk wtf28:hphMX6::ade6-* | this work |
| SZY1345 |  | *Sk* | *h90, ura4*Δ*::natMX4, lys1*Δ*::kanMX4, ade6*Δ*::hphMX6, rec12*Δ*::ura4+, Sk wtf4*Δ*::kanMX4* (truncated drugS) | this work |
| SZY1346 |  | *Sk* | *h90, ura4*Δ*::natMX4, lys1*Δ*::kanMX4, ade6*Δ*::hphMX6, rec12*Δ*::ura4+, Sk wtf4*Δ*::kanMX4* (truncated drugS) | this work |
| SZY1351 |  | *Sp* | *h-, lys4-95, ade6-::Sk wtf28(M1X, M12X):hphMX6::ade6-* | this work |
| SZY1391 |  | hybrid with *Sk* karyotype | hybrid chr3, *Sk* chr1 and chr2. *h90, rec12*∆*::ura4+, his5*∆*::natMX4, ura4-294*, *Sp* *wtf13*∆::*kanMX4* | this work |
| SZY1392 |  | hybrid with *Sk* karyotype | hybrid chr3, *Sk* chr1 and chr2. *h90, rec12*∆*::ura4+, his5*∆*::natMX4, ura4-294*, *Sp wtf13*∆::*kanMX4* | this work |
| SZY1393 |  | hybrid with *Sk* karyotype | hybrid chr3, *Sk* chr1 and chr2. *h90, rec12*∆*::ura4+, his5*∆*::natMX4, ura4-294*, *Sp wtf13*∆::*kanMX4* | this work |
| SZY1394 |  | hybrid with *Sk* karyotype | hybrid chr3, *Sk* chr1 and chr2. *h90, rec12*∆*::ura4+, his5*∆*::natMX4, ura4-294*, *Sp wtf13*∆::*kanMX4* | this work |
| SZY1404 |  | *Sk* | *h90, ura4*Δ*::natMX4, ade6-::Sp wtf13::hphMX6::ade6-* | Bravo Núñez et al 2018 |
| SZY1485 |  | *Sk* | *h90, ura4*∆*::natMX4, wtf4*∆*::kanMX4* *(truncated drugS),*  *ade6-::Sk wtf4:kanMX4::ade6-* | this work |
| SZY1486 |  | *Sk* | *h90, ura4*∆*::natMX4, wtf4*∆*::kanMX4* *(truncated drugS),*  *ade6-::Sk wtf4:kanMX4::ade6-* | this work |
| SZY1496 |  | *Sk* | *h90, his5*Δ*::natMX4, ade6-::Sp wtf13:kanMX4::ade6-* | Bravo Núñez et al 2018 |
| SZY1516 |  | *Sk* | *h90, his5*Δ*::natMX4, ade6-::kanMX4::ade6-* | Bravo Núñez et al 2020 |
| SZY1518 |  | *Sk* | *h90, his5*∆*::natMX4, ade6-::hphMX6::ade6-* | Bravo Núñez et al 2018 |
| SZY1535^#^ |  | *Sk* | *h90, ura4*∆*::natMX4, wtf4*∆*::kanMX4* (truncated drugS)*,*  *ade6-::Sp wtf13:hphMX6::ade6-,* Ura+ | this work |
| SZY1537^#^ |  | *Sk* | *h90, ura4*∆*::natMX4, wtf4*∆*::kanMX4* (truncated drugS)*,*  *ade6-::Sp wtf13:hphMX6::ade6-,* Ura+ | this work |
| SZY1583 |  | *Sp* | *h?, leu1-32, ura4-D18, ade6-::Sk wtf4:kanMX4::ade6-, rec12-171::ura4+* | this work |
| SZY1585 |  | *Sp* | *h?, lys4-95, ade6-::Sk wtf28:hphMX6::ade6-, rec12-171::ura4+* | this work |
| SZY1595 |  | hybrid with *Sk* karyotype | hybrid chr3, *Sk* chr1 and chr2*. h90, rec12*∆*::ura4+, his5*∆*::kanMX4, ura4-x* | this work |
| SZY1699 |  | hybrid with *Sk* karyotype | hybrid chr3*, Sk* chr1 and chr2*. h90, rec12*∆*::ura4+, his5*∆*::kanMX4, ura4-294, Sp wtf4*∆ | this work |
| SZY1735 |  | *Sp* | *h-, lys4-95, sgo1*∆*::hphMX6* | this work |
| SZY1736 |  | *Sp* | *h90, leu1-32, ura4-D18, sgo1*∆*::hphMX6* | this work |
| SZY1770 |  | *Sp* | *h90, leu1-32, ura4-D18, rec12-171::ura4+,*  *ade6-::kanMX4::ade6-* | this work |
| SZY1981 |  | hybrid with *Sk* karyotype | hybrid chr3, *Sk* chr1 and chr2*. h90, rec12*∆*::ura4+, his5*∆*::natMX4, ura4-x, Sp wtf4*∆ | this work |
| SZY1982 |  | hybrid with *Sk* karyotype | hybrid chr3*, Sk* chr1 and chr2*. h90, rec12*∆*::ura4+, his5*∆*::natMX4, ura4-x, Sp wtf4*∆ | this work |
| SZY1994 |  | *Sp* | *h?, lys4-95, ade6-::hphMX6::ade6-, rec12-171::ura4+* | this work |
| SZY2008 |  | hybrid with *Sk* karyotype | hybrid chr3, *Sk* chr1 and chr2*. h90, rec12*∆*::ura4+, his5*∆*::natMX4, ura4-294, Sp wtf4*∆*,*  *Sp wtf13*∆*::kanMX4* | this work |
| SZY2010 |  | hybrid with *Sk* karyotype | hybrid chr3*, Sk* chr1 and chr2*. h90, rec12*∆*::ura4+, his5*∆*::natMX4, ura4-294, Sp wtf4*∆*,*  *Sp wtf13*∆*::kanMX4* | this work |
| SZY2036 |  | *Sp* | *h90, leu1-32, ura4-D18, ade6-::Sk wtf4:kanMX4::ade6-, sgo1*∆*::natMX4* | this work |
| SZY2038 |  | *Sp* | *h-, lys4-95, ade6-::Sk wtf28:hphMX6::ade6-, sgo1*∆*::natMX4* | this work |
| SZY2111 |  | CBS5680 | *h90, ade6*Δ*::hphMX6* | this work |
| SZY2153 |  | JB844 | *h90, lys1*Δ::*kanMX4* | this work |
| SZY2212 |  | JB1172 | *h90, lys1*Δ::*kanMX4* | this work |
| SZY2213 |  | CBS5680 | *h90, lys1*Δ::*kanMX4* | this work |
| SZY2214 |  | JB844 | *h?, ade6-::hphMX6::ade6-* | this work |
| SZY2221 |  | JB1172 | *h90, ade6*Δ*::hphMX6* | this work |
| SZY2222 |  | JB844 | *h90, ade6*Δ*::hphMX6* | this work |
| SZY2225 |  | *Sp* | *h-, lys4-95, ade6-::*FY29033 *wtf35::kanMX4::ade6-* | Bravo Núñez et al 2020 |
| SZY2397 |  | *Sp* | *h+, lys1-37, rec12-169::3HA6His-kanMX6, ura4*Δ*::natMX4* | this work |
| SZY2398 |  | *Sp* | *h+, lys1-37, rec12-169::3HA6His-kanMX6, ura4*Δ*::natMX4* | this work |
| SZY2430 |  | *Sp* | *h90, leu1-32, ura4-D18, ade6-::*FY29033 *wtf36::hphMX6::ade6-* | Bravo Núñez et al 2020 |
| SZY2431 |  | *Sk* | *h90, ura4*Δ*::natMX4, ade6-::*FY29033 *wtf36::hphMX6::ade6-* | Bravo Núñez et al 2020 |
| SZY2464 |  | *Sp* | *h90, ade6*Δ*::hphMX6* | this work |
| SZY2479 |  | *Sp* | *h-, lys4-95, moa1*∆*::natMX4* | this work |
| SZY2481 |  | *Sp* | *h90, leu1-32, ura4-D18, moa1*∆*::natMX4* | this work |
| SZY2494 |  | *Sp* | *h90, leu1-32, ura4-D18, moa1*∆*::natMX4, ade6-::kanMX4::ade6-* | this work |
| SZY2496 |  | *Sp* | *h90, leu1-32, ura4-D18, moa1*∆*::natMX4, ade6-::Sk wtf4::kanMX4::ade6-* | this work |
| SZY2498 |  | *Sp* | *h-, lys4-95, moa1*∆*::natMX4, ade6-::Sk wtf28::hphMX6::ade6-* | this work |
| SZY2500 |  | *Sp* | *h90, leu1-32, ura4-D18, sgo1*∆*::hphMX6, ade6-::kanMX4::ade6-* | this work |
| SZY2515 |  | *Sp* | *h-, lys4-95, moa1*∆*::natMX4, ade6-::hphMX6::ade6-* | this work |
| SZY2517 |  | *Sp* | *h90, leu1-32, ura4-D18, rec10*∆*::natMX4* | this work |
| SZY2519 |  | *Sp* | *h-, lys4-95, rec10*∆*::natMX4* | this work |
| SZY2521 |  | *Sp* | *h-, lys4-95, sgo1*∆*::hphMX6, ade6-::natMX4::ade6-* | this work |
| SZY2523 |  | *Sp* | *h-, lys4-95, ade6-::Sk wtf4::kanMX4::ade6-* | this work |
| SZY2532 |  | *Sp* | *h90, ura4-D18, ade6-::Sk wtf28::hphMX6::ade6-* | this work |
| SZY2538 |  | *Sp* | *h90*, *lys1*Δ::*kanMX4* | this work |
| SZY2542 |  | *Sp* | *h90, leu1-32, ura4-D18, rec10*Δ*::natMX4, ade6-::kanMX4::ade6-* | this work |
| SZY2544 |  | *Sp* | *h90, leu1-32, ura4-D18, rec10*Δ*::natMX4, ade6-::Sk wtf4::kanMX4::ade6-* | this work |
| SZY2546 |  | *Sp* | *h-, lys4-95, rec10*Δ*::natMX4, ade6-::hphMX6::ade6-* | this work |
| SZY2548 |  | *Sp* | *h-, lys4-95, rec10*Δ*::natMX4, ade6-::Sk wtf28::hphMX6::ade6-* | this work |
| SZY2625 |  | *Sp* | *h90, leu1-32, ura4-D18, ade6*-::*kanMX4*::*ade6-, his5*Δ::*ade6+* | this work |
| SZY2628 |  | *Sp* | *h90, leu1-32, ura4-D18, ade6-::Sk wtf4*:*kanMX4*::*ade6-, his5*Δ*::ade6+* | this work |
| SZY2704 |  | JB4 | *h?, ade6-::hphMX6::ade6-* | this work |
| SZY2706 |  | JB4 | *h?, lys1-::kanMX4::lys1-* | this work |
| SZY2712 |  | JB878 | *h?, ade6-::hphMX6::ade6-* | this work |
| SZY2714 |  | JB878 | *h?, lys1-::kanMX4::lys1-* | this work |
| SZY2718 |  | JB899 | *h?, ade6-::hphMX6::ade6-* | this work |
| SZY2720 |  | JB899 | *h?, lys1-::kanMX4::lys1-* | this work |
| SZY2722 |  | JB910 | *h?, ade6-::hphMX6::ade6-* | this work |
| SZY2724 |  | JB910 | *h?, lys1-::kanMX4::lys1-* | this work |
| SZY2750 |  | *Sp* | *h90, ade6-::hphMX6::ade6-* | this work |
| SZY2752 |  | *Sp* | *h90, lys1-::kanMX4::lys1-* | this work |
| SZY2756 |  | *Sk* | *h90, lys1-::kanMX4::lys1-* | this work |
| SZY2824 |  | JB848 | *h?, ade6-::hphMX6::ade6-* | this work |
| SZY2826 |  | JB848 | *h?, lys1-::kanMX4::lys1-* | this work |
| SZY2828 |  | JB852 | *h?, ade6-::hphMX6::ade6-* | this work |
| SZY2830 |  | JB852 | *h?, lys1-::kanMX4::lys1-* | this work |
| SZY2832 |  | JB854 | *h?, ade6-::hphMX6::ade6-* | this work |
| SZY2834 |  | JB854 | *h?, lys1-::kanMX4::lys1-* | this work |
| SZY2838 |  | JB862 | *h?, ade6-::hphMX6::ade6-* | this work |
| SZY2840 |  | JB862 | *h?, lys1-::kanMX4::lys1-* | this work |
| SZY2842 |  | JB873 | *h?, ade6-::hphMX6::ade6-* | this work |
| SZY2844 |  | JB873 | *h?, lys1-::kanMX4::lys1-* | this work |
| SZY2850 |  | JB879 | *h?, ade6-::hphMX6::ade6-* | this work |
| SZY2852 |  | JB879 | *h?, lys1-::kanMX4::lys1-* | this work |
| SZY2916 |  | JB840 | *h?, ade6-::hphMX6::ade6-* | this work |
| SZY2918 |  | JB840 | *h?, lys1-::kanMX4::lys1-* | this work |
| SZY3076 |  | JB844 | *h?, lys1-::kanMX4::lys1-* | this work |
| SZY3113 |  | *Sp* | *h-, lys4-95, ade6-::hphMX6::ade6-, ura4-::kanMX4::ura4-* | this work |
| SZY3181 |  | *Sp* | *h-, lys4-95, ade6-::Sk wtf4:natMX4::ade6-, ura4-::*FY29033 *wtf35:kanMX4::ura4-* | this work |
| SZY3182 |  | *Sp* | *h-, lys4-95, ade6-::Sk wtf4:natMX4::ade6-, ura4-::*FY29033 *wtf35:kanMX4::ura4-* | this work |
| SZY3195 |  | *Sp* | *h90, leu1-32, ade6-::*FY29033 *wtf36:hphMX6::ade6-, ura4-::Sk wtf28:kanMX4::ura4-* | this work |
| SZY3197 |  | *Sp* | *h90, leu1-32, ade6-::*FY29033 *wtf36:hphMX6::ade6-, ura4-::Sk wtf28:kanMX4::ura4-* | this work |
| SZY3233 |  | JB939 | *h?, ade6-::hphMX6::ade6-* | this work |
| SZY3235 |  | JB939 | *h?, lys1-::kanMX4::lys1-* | this work |
| SZY3236 |  | JB853 | *h?, ade6-::hphMX6::ade6-* | this work |
| SZY3238 |  | JB853 | *h?, lys1-::kanMX4::lys1-* | this work |
| SZY3354 |  | *Sp* | *h-, leu1-32, ura4-D18, rec8::ura4+, ade6-::kanMX4::ade6-* | this work |
| SZY3356 |  | *Sp* | *h-, leu1-32, ura4-D18, rec8::ura4+, ade6-::Sk wtf4:kanMX4::ade6-* | this work |
| SZY3399 |  | *Sp* | *h90, leu1-32, ade6-::natMX4::ade6-, ura4-::kanMX4::ura4-* | this work |
| SZY3509 |  | *Sk* | *h90, lys4*Δ*::kanMX4, ade6-::natMX4::ade6-* | Bravo Núñez et al 2020 |
| SZY3511 |  | *Sk* | *h90, lys4*Δ*::kanMX4,* *ade6-::*FY29033 *wtf36:natMX4::ade6-* | this work |
| SZY3512 |  | *Sk* | *h90, lys4*Δ*::kanMX4,* *ade6-::*FY29033 *wtf36:natMX4::ade6-* | this work |
| SZY3539 |  | *Sk* | *h90, wtf4*Δ*::kanMX4* (truncated drugS), *ura4-::*FY29033 *wtf35:kanMX4::ura4-* | this work |
| SZY3581 |  | *Sk* | *h90, wtf4*Δ*::kanMX4* (truncated drugS)*, ura4-::*FY29033 *wtf35:kanMX4::ura4-, ade6-::Sp wtf13:hphMX6::ade6-* | this work |
| SZY3582 |  | *Sk* | *h90, wtf4*Δ*::kanMX4* (truncated drugS)*, ura4-::*FY29033 *wtf35:kanMX4::ura4-, ade6-::Sp wtf13:hphMX6::ade6-* | this work |
| SZY3601 |  | *Sp* | *h?, lys4-95, ura4-D18, rec8::ura4+, ade6-::hphMX6::ade6-* | this work |
| SZY3603 |  | *Sp* | *h?, lys4-95, ura4-D18, rec8::ura4+, ade6-::Sk wtf28:hphMX6::ade6-* | this work |
| SZY3621 |  | *Sk* | *h90, ura4*Δ*::natMX4, ade6-::*FY29033 *wtf35:hphMX6::ade6-* | this work |
| SZY3654 |  | *Sk* | *h90, ade6-::*FY29033 *wtf36:natMX4::ade6-, rec12*∆*::ura4+* | this work |
| SZY3656 |  | *Sk* | *h90, ade6-::natMX4::ade6-, rec12*∆*::ura4+* | this work |
| SZY3709 |  | JB942 | *h?, ade6-::hphMX6::ade6-* | this work |
| SZY3721 |  | JB942 | *h?, lys1-::kanMX4::lys1-* | this work |
| SZY3834 |  | *Sp* | *h90, leu1-32, rec12-117, ura4-x, lys1-37* | Bravo Núñez et al 2020 |
| SZY3850 |  | *Sk* | *h90, ura4-::kanMX4::ura4-, ade6-::hphMX6::ade6-, rec12*∆*::ura4+* | this work |
| SZY3855 |  | *Sk* | *h90, wtf4*∆*::kanMX4* (truncated drugS), *ura4-::*FY29033 *wtf35:kanMX4::ura4-, ade6-::Sp wtf13:hphMX6::ade6-, rec12*∆*::ura4+* | this work |
| SZY3909 |  | JB844 | *h90, ura4-D18, rec12*∆*::ura4+, ade6*∆*::hphMX6, his5*∆*::natMX4* | this work |
| SZY3910* |  | JB844 | *h90, (ura4-D18?), rec12*∆*::ura4+, lys1*∆*::kanMX4* | this work |
| SZY3911* |  | JB844 | *h90, (ura4-D18?), rec12*∆*::ura4+, lys1*∆*::kanMX4* | this work |
| SZY3949 |  | CBS5680 | *h90, ade6*Δ*::hphMX6, ura4-D18* | this work |
| SZY3954 |  | *Sk* | *h90, ade6*Δ*::hphMX6, ura4-::kanMX4::ura4-* | Bravo Núñez et al 2020 |
| SZY3995 |  | CBS5680 | *h90, ade6*Δ*::hphMX6, ura4-D18, rec12*Δ*::ura4+* | this work |
| SZY4065 |  | CBS5680 | *h90, lys1*Δ*::kanMX4, ura4-D18, rec12*Δ*::ura4+* | this work |
| SZY4066 |  | CBS5680 | *h90, lys1*Δ*::kanMX4, ura4-D18, rec12*Δ*::ura4+* | this work |
