# Supplementary material for: Atypical meiosis can be adaptive in outcrossed Schizosaccharomyces pombe due to wtf meiotic drivers
Source: eLife. 2020 Aug 13;9:e57936. doi: 10.7554/eLife.57936 (PMC7426094; doi:10.7554/eLife.57936)
Supplement: Supplementary file 2. [file elife-57936-supp2.docx]

| **Plasmids** | **short description** | **reference** |
| --- | --- | --- |
| pFA6 | contains kanMX4 | Wach et al 1994 |
| pAG25 | contains natMX4 | Goldstein and McCusker 1999 |
| pAG32 | contains hphMX6 | Goldstein and McCusker 1999 |
| pMZ379 | contains Cas9 | Rodríguez López et al 2016 |
| pSZB188 | derivative of pFA6 that integrates at *ade6*, yielding *ade6*- | Nuckolls et al 2017 |
| pSZB189 | pSZB188 with *Sk wtf4* cloned into SacI site | Nuckolls et al 2017 |
| pSZB254 | pSZB188 with *Sk wtf28* into SacI site | Nuckolls et al 2017 |
| pSZB331 | derivative of pFA6 that integrates at *ura4*, yielding *ura*- | Bravo Núñez et al 2020 |
| pSZB332 | derivative of pFA6 that integrates at *ura4*, yielding *ura*- | Bravo Núñez et al 2020 |
| pSZB386 | derivative of pAG32 that integrates at *ade6*, yielding *ade*- | Bravo Núñez et al 2018 |
| pSZB412 | pSZB386 with *Sk wtf28* cloned into SacI site | this work |
| pSZB414 | pSZB386 with *Sk wtf28poison* cloned into the SacI site | this work |
| pSZB570 | derivative of pMZ379 containing the *Sp wtf4* gRNA | this work |
| pSZB718 | pSZB332 with *Sk wtf28* cloned into SacI site | this work |
| pSZB722 | derivative of pAG25 that integrates at *ade6*, yielding *ade*- | Bravo Núñez et al 2020 |
| pSZB723 | derivative of pAG25 that integrates at *ade6*, yielding *ade*- | Bravo Núñez et al 2020 |
| pSZB788 | pSZB188 with FY29033 *wtf35* cloned into SacI site | Bravo Núñez et al 2020 |
| pSZB816 | derivative of pFA6 that integrates at *lys1*, yielding *lys*- | this work |
| pSZB849 | derivative of pAG25 that integrates at *ade6*, yielding *ade*- | Bravo Núñez et al 2020 |
| pSZB923 | pSZB849 with *Sk wtf4* into SacI site | this work |
| pSZB1001 | pSZB331 with FY29033 *wtf35* into SacI site | this work |
| pSZB1060 | pSZB849 with FY29033 *wtf36* into SacI site | this work |
