# Supplementary material for: Atypical meiosis can be adaptive in outcrossed Schizosaccharomyces pombe due to wtf meiotic drivers
Source: eLife. 2020 Aug 13;9:e57936. doi: 10.7554/eLife.57936 (PMC7426094; doi:10.7554/eLife.57936)
Supplement: Supplementary file 3. — Column 1 lists the wtf gene cloned into each vector. Column 2 denotes the isolate from which each wtf was cloned. The DNA templates and oligos used in the PCR reactions to amplify the wtf alleles are shown in columns 3 and 4, respectively. We digested each of the amplified fragments with the enzymes reported in column 5 and then integrated into the target site listed in column 6. The strain number of each of the plasmids that we generated is reported in column 7. The description of each plasmid can be found in Supplementary file 2. [file elife-57936-supp3.docx]

| ***wtf* allele** | ***S. pombe*  isolate** | **DNA**  **template** | **PCR oligos used** | **PCR fragment digested with  restriction enzyme** | **target site** | **Resulting  plasmid number** |
| --- | --- | --- | --- | --- | --- | --- |
| *wtf28* | *Sk* | pSZB254 | 733+651 | SacI | SacI site of pSZB386 | pSZB412 |
| *wtf28* | *Sk* | SZY661 | 733+651 | SacI | SacI site of pSZB332 | pSZB718 |
| *wtf35* | FY29033 | pSZB788 | 1036+1349 | SacI | SacI site of pSZB331 | pSZB1001 |
| *wtf36* | FY29033 | SZY1150 | 1351+1593 | SacI | SacI site of pSZB849 | pSZB1060 |
