# Supplementary material for: Atypical meiosis can be adaptive in outcrossed Schizosaccharomyces pombe due to wtf meiotic drivers
Source: eLife. 2020 Aug 13;9:e57936. doi: 10.7554/eLife.57936 (PMC7426094; doi:10.7554/eLife.57936)
Supplement: Supplementary file 4. [file elife-57936-supp4.docx]

| **Oligo number** | **Sequence (5’🡪3’)** |
| --- | --- |
| AO1112 | TAACGCCGCCATCCAGTGTCG |
| AO638 | GTATTCTGGGCCTCCATGTCG |
| M13F-41 | GGTTTTCCCAGTCACGAC |
| 34 | GAAGCTTAGCTACAAATCCCACTGGC |
| 37 | CGGTTCAAACTCCCTCTCAGCG |
| 471 | ATTATTGAGCTCACTACGATGCAATGGCATAGCATGGC |
| 543 | CCGTCTCGCTTCCCTTTCTCAAATGTTG |
| 548 | GGATTTTGGGTCTCTAGAGCATAAGACAC |
| 574 | CCAGGCAACATCCATTCTCATCAGATGAGG |
| 651 | AATATAGAGCTCCCGAAGTATCATATCAACGTAGTACACCATG |
| 733 | AATATAGAGCTCCTTCTTATTCACCCCAACTTAGATTTCCTTATGCATC |

| 953 | CATTCAATAATAGATTGTTTTTAAGAATAGAAGAATAAATATTATCCCTTGAGGTCGTCTTAGGATGAATTGAG |
| --- | --- |
| 954 | CTCAATTCATCCTAAGACGACCTCAAGGGATAATATTTATTCTTCTATTCTTAAAAACAATCTATTATTGAATG |

| 1036 | ATTATT GAGCTCCATACTCCTCTTGTTTAGGCGACATTCG |
| --- | --- |
| 1048 | CAGCTTCGGTAGAAACTTTGCGTCAAAATC |
| 1049 | GTGTCACCTAAATCGTATGTGGCGACAAACGAAACAGCGAAATCAAGAATA |
| 1050 | TATTCTTGATTTCGCTGTTTCGTTTGTCGCCACATACGATTTAGGTGACAC |
| 1051 | ACTGGAGCGACAACCACATTAATAAAGCAAAATACGACTCACTATAGGGAG |
| 1052 | CTCCCTATAGTGAGTCGTATTTTGCTTTATTAATGTGGTTGTCGCTCCAGT |
| 1053 | TTCGCTGTTGACTTAGAAATCCCTGCCTAA |
| 1058 | AGTTGCATACATTACCAAAGAAGCGTTGAATC |
| 1059 | GGGTCCAAGTTTCAAAGAAGCCTAATGAGTTAG |
| 1060 | AATTTACTCTAACCATACCAGAACGTCCAGGT |
| 1061 | CATGCACGTTACCACATCAAAAGTAGTAGGAA |
| 1069 | GAAGCAAAAGGAGGTATTGTTTGATGGGATG |
| 1077 | TCTGTCCCTTCGTACGTATCGGATGGA |
| 1108 | GCTTGGCAGGTAATTGAGAAAGCG |
| 1120 | CCAACCCATATCATAGAGCCCCTTTTATATGC |
| 1138 | GCACTTTAGACTTGTTTATTTCTGTTCCCAAACTGCGTAGCTGACATGACACTGAATTTC |
| 1139 | AAATTCAGTGTCATGTCAGCTACGCAGTTTGGGAACAGAAATAAACAAGTCTAAAGTGCC |
| 1187 | GCGGGGACGAGGCAAGCTAAGCCATAAATGCAATCTATTGTTTTTTTTCGCTTAATG |
| 1189 | GTACGATTATATTCCAGTGTCAGTTAGAGATTAGGGAGACCGGCAGATCCG |
| 1190 | CGGATCTGCCGGTCTCCCTAATCTCTAACTGACACTGGAATATAATCGTAC |
| 1191 | GAAATATACGATTGAACCACGGAATATGCGG |
| 1192 | GTTAGTGGTTGGAATCAAATTTCCAGCATACC |
| 1194 | GCTATCACTATTGTCTTGACGAAGTATTGCACC |
| 1206 | CTAACTCATTgttttagagctagaaatagcaagttaaaataa |
| 1207 | TGAATGGACATTCTTCGGTACAGGTTATGTTTTTTGGCAACA |
| 1224 | ACTTGACCGAAAAGCACTTGACTCG |
| 1225 | GTGTCACCTAAATCGTATGTGAGGACTTGCTGATGTAAACAAACCTGT |
| 1226 | ACAGGTTTGTTTACATCAGCAAGTCCTCACATACGATTTAGGTGACAC |
| 1227 | GCCTTGATTAGGTTCTTTTTTTCTTCCTCTAATACGACTCACTATAGGGAG |
| 1228 | CTCCCTATAGTGAGTCGTATTAAGAGGAAGAAAAAAAGAACCTAATCAAGGC |
| 1229 | CCTATCAACTCGCCTAGTCTTGCATTTTT |
| 1230 | CCCTTTTCTAACTTCTTAGTGCGTGC |
| 1231 | CCTTGAAATTCTGTTCCTCATACAGTGC |
| 1349 | ATTATTGAGCTCTCATTAGTTGATGATAACAGTCAATTAGTGAACTGC |
| 1351 | ATTATTGAGCTCTAACAAGGCGGAACAACGAGTTTTCC |
| 1593 | ATTATTGAGCTCCCAAGAAGGGAATGTTCATGCTATCCATG |
| 1673 | GCGCAGTGAATGTAAAACGAAATTCAGGG |
| 1674 | CGCTGACTTTATCCACTTTTCTTTGTCGGCC |
| 1675 | CATTAAGCGAAAAAAAACAATAGATTGCATTTATGGCCACATACGATTTAGGTGACAC |
| 1701 | GTTATGTAAGTTGCAGTTCTGCATCCCAGTAAGG |
| **Oligo number** | **Sequence (5’🡪3’)** |
| 1702 | GGTACACAAACGGTCACTTGGTAGTACCTC |
| 1703 | GAATTCGACACGTCCAGCTTTACAAGAC |
| 1704 | TGGGTATTCTTGATGTTTGAAACTAACCG |
| 1723 | CTATTTTCGTTTATCTTTCCTGTTTATAGG |
| 1724 | GTGTCACCTAAATCGTATGTGGGAATAATAAATAGGATTAAATACGTAAAACGG |
| 1725 | CCGTTTTACGTATTTAATCCTATTTATTATTCCCACATACGATTTAGGTGACAC |
| 1726 | GCCAATATCAATAAATTTGATACTATAGGTTCAATACGACTCACTATAGGGAG |
| 1727 | CTCCCTATAGTGAGTCGTATTGAACCTATAGTATCAAATTTATTGATATTGGC |
| 1728 | CTCGTGTTCGTGAACTGGAGATG |
| 1729 | GAACCATTTAACTTCGGAGAATATTGAGACTCCAG |
| 1730 | CCATCAATAGTAGCAGATTTGCTAGATGGATTG |
| 1731 | GTCAGCTGCGGTGTGTTCAGTTTC |
| 1732 | CTTATAAGGGTGCTCAGTCTCCCAATGC |
| 2088 | GCTTTGCCTCTGGGATGTCGTTTG |
| 2089 | GATAAATTGCCGATGGAGTCGTTG |
| 2415 | CTACAAAGATCGTTATGTTTATCGGCACTTTGCATCGGCC |
| 2416 | CTCTCGCTGAATTCCCCAATGTCAAGCACTTCCGGAATC |
| 2417 | CCGTCAGCCAGTTTAGTCTGACCATCTCATCTGTAACATC |
| 2418 | GAAAATATTGTTGATGCGCTGGCAGTGTTCCTGCG |
