# Supplementary material for: Atypical meiosis can be adaptive in outcrossed Schizosaccharomyces pombe due to wtf meiotic drivers
Source: eLife. 2020 Aug 13;9:e57936. doi: 10.7554/eLife.57936 (PMC7426094; doi:10.7554/eLife.57936)
Supplement: Transparent reporting form [file elife-57936-transrepform.docx]

***eLife’s* transparent reporting form**

We encourage authors to provide detailed information *within their submission* to facilitate the interpretation and replication of experiments. Authors can upload supporting documentation to indicate the use of appropriate reporting guidelines for health-related research (see EQUATOR Network), life science research (see the BioSharing Information Resource), or the ARRIVE guidelines for reporting work involving animal research. Where applicable, authors should refer to any relevant reporting standards documents in this form.

If you have any questions, please consult our Journal Policies and/or contact us:

editorial@elifesciences.org.

# Sample-size estimation

- You should state whether an appropriate sample size was computed when the study was being designed
- You should state the statistical method of sample size computation and any required assumptions
- If no explicit power analysis was used, you should describe how you decided what sample (replicate) size (number) to use

Please outline where this information can be found within the submission (e.g., sections or figure legends), or explain why this information doesn’t apply to your submission:

We used power analyses to determine the sample sizes we assayed for the recombination data presented in Figure 2—figure supplement 7 and Figure 3—figure supplement 2. For the other experiments, we did not use power analyses to determine the sample sizes we assayed. The sample sizes we used for crosses are presented in:

- Figure 1 are shown in Figure 1—figure supplement 4 and 5
- Figure 1—figure supplement 3 are shown in Figure 1—figure supplement 6.
- Figure 2, Figure 2—figure supplement 1, Figure 2—figure supplement 2, and Figure 2—figure supplement 3 are shown in Figure 2—figure supplement 5 and 6
- Figure 3 and Figure 3—figure supplement 1 are shown in Figure 3—figure supplement 3 and 4
- Figure 4 and figure 4—figure supplement 2 are shown in Figure 4—figure supplement 6 and 7
- Figure 5 are shown in Figure 5—figure supplement 1
- Figure 6 and Figure 6—figure supplement 1 are shown in Figure 6—figure supplement 3 and 4.

All of these supplement files also contained the number of progeny for characterizing meiotic drive, disomy, and fertility in *S. pombe* and *S. kambucha*.

The sample sizes we used to determine the type of G418R HygR spores are reported in Methods-*Chromosome loss assay*.

# Replicates

- You should report how often each experiment was performed
- You should include a definition of biological versus technical replication
- The data obtained should be provided and sufficient information should be provided to indicate the number of independent biological and/or technical replicates
- If you encountered any outliers, you should describe how these were handled
- Criteria for exclusion/inclusion of data should be clearly stated
- High-throughput sequence data should be uploaded before submission, with a private link for reviewers provided (these are available from both GEO and ArrayExpress)

Please outline where this information can be found within the submission (e.g., sections or figure legends), or explain why this information doesn’t apply to your submission:

We assayed at least two independent (biological and technical replicates) diploids for each experiment. The absolute number of diploids assayed for individual crosses (for fertility and allele transmission) can be found in Figure 1—figure supplement 4-6; Figure 2—figure supplement 5 and 6; Figure 3—figure supplement 3 and 4; Figure 4—figure supplement 6 and 7; Figure 5—figure supplement 1; and Figure 6—figure supplement 3 and 4

We did not remove outliers, and we excluded certain samples (those that were not heterozygous) from our analyses (Methods-Fertility and allele transmission (Bravo Núñez et al 2020)).

# Statistical reporting

- Statistical analysis methods should be described and justified
- Raw data should be presented in figures whenever informative to do so (typically when N per group is less than 10)
- For each experiment, you should identify the statistical tests used, exact values of N, definitions of center, methods of multiple test correction, and dispersion and precision measures (e.g., mean, median, SD, SEM, confidence intervals; and, for the major substantive results, a measure of effect size (e.g., Pearson's r, Cohen's d)
- Report exact p-values wherever possible alongside the summary statistics and 95% confidence intervals. These should be reported for all key questions and not only when the p-value is less than 0.05.

Please outline where this information can be found within the submission (e.g., sections or figure legends), or explain why this information doesn’t apply to your submission:

We used G-tests to determine changes in allele transmission and recombination frequencies, and Wilcoxon tests to determine changes in spore viability (fertility). We highlighted values where *p* was less than 0.05, although they were often much smaller. This can be found in the legends of Figure 1, Figure 2, Figure 2—figure supplement 1, Figure 2—figure supplement 2, Figure 2—figure supplement 3, Figure 3, Figure 3—figure supplement 1, Figure 4, Figure 4—figure supplement 2, Figure 5, Figure 6, and Figure 6—figure supplement 1.

Actual p-values for all crosses shown in the main and supplemental figures are listed in Figure 1—figure supplement 4 and 5; Figure 2—figure supplement 5 and 6; Figure 3—figure supplement 3 and 4; Figure 4—figure supplement 6 and 7; Figure 5—figure supplement 1; and Figure 6—figure supplement 3 and 4. These figures/files also include all N values.

Figure 1—figure supplement 6 contains the N values for Figure 1—figure supplement 3.

 (For large datasets, or papers with a very large number of statistical tests, you may upload a single table file with tests, Ns, etc., with reference to sections in the manuscript.)

# Group allocation

- Indicate how samples were allocated into experimental groups (in the case of clinical studies, please specify allocation to treatment method); if randomization was used, please also state if restricted randomization was applied
- Indicate if masking was used during group allocation, data collection and/or data analysis

Please outline where this information can be found within the submission (e.g., sections or figure legends), or explain why this information doesn’t apply to your submission:

Samples were determined as experimental or control groups depending on the genotype of the yeast used for each experiment.

# Additional data files (“source data”)

- We encourage you to upload relevant additional data files, such as numerical data that are represented as a graph in a figure, or as a summary table
- Where provided, these should be in the most useful format, and they can be uploaded as “Source data” files linked to a main figure or table
- Include model definition files including the full list of parameters used
- Include code used for data analysis (e.g., R, MatLab)
- Avoid stating that data files are “available upon request”

Please indicate the figures or tables for which source data files have been provided:

All the raw data is reported in the supplements for each Figure.
